# Bedside Ultrasound of Quadriceps to Predict Rehospitalization and Functional Decline in Hospitalized Elders

**DOI:** 10.3389/fmed.2017.00122

**Published:** 2017-07-31

**Authors:** Ana Clara Guerreiro, Ana Claudia Tonelli, Roman Orzechowski, Roberta Rigo Dalla Corte, Emilio Hideyuki Moriguchi, Renato Bandeira de Mello

**Affiliations:** ^1^Geriatric Medicine Residency Program, Hospital de Clínicas de Porto Alegre, Porto Alegre, Brazil; ^2^Division of Internal Medicine, Hospital de Clínicas de Porto Alegre, Porto Alegre, Brazil; ^3^Health School, Universidade do Vale do Rio dos Sinos, São Leopoldo, Brazil; ^4^Department of Internal Medicine, School of Medicine, Universidade Federal do Rio Grande do Sul, Porto Alegre, Brazil; ^5^Postgraduate Studies Program in Cardiology, School of Medicine, Universidade Federal do Rio Grande do Sul, Porto Alegre, Brazil; ^6^Postgraduate Studies Program in Endocrinology, School of Medicine, Universidade Federal do Rio Grande do Sul, Porto Alegre, Brazil

**Keywords:** hospitalization, functional decline, elderly, point of care ultrasound, comprehensive geriatric assessment

## Abstract

**Objective:**

To evaluate the capacity of total anterior thigh thickness, quadriceps muscle thickness, and quadriceps contractile index, all measured by bedside ultrasound, to predict rehospitalization, functional decline, and death in elderly patients 3 months after hospital discharge. To evaluate intra and interobserver reproducibility of the dominant thigh evaluation method by point of care ultrasound.

**Methods:**

Cohort study of patients aged 65 years or more admitted to a medium complexity unit in a teaching hospital in southern Brazil. Comprehensive geriatric assessment and ultrasound evaluation of the dominant thigh of each participant were performed. After 3 months of hospital discharge, telephone contact was made to evaluate the outcomes of rehospitalization or death and functional decline—assessed by the 100 points Barthel scale and defined as a decrease of five or more points.

**Results:**

100 participants were included. There was no statistically significant difference between intraobserver measurements in the GEE method analysis (*p* > 0.05), and the mean bias obtained in Bland–Altman plots was close to zero in all four analyses performed, suggesting good intra and interobserver agreement. There was a significant correlation between the echographic measurements (quadriceps thickness and contractile index) and gait speed, timed up and go, and handgrip tests. There was a significant association between contractile index (quadriceps thickness over total anterior thigh thickness multiplied by 100) lower than 60% and functional decline (relative risk 1.35; CI 95% 1.10–1.65; *p* = 0.003) as well as between the thickness of the quadriceps and rehospitalization or death, in both individuals with preserved walking capacity and in bedridden elders (relative risk 1.34; CI 95% 1.02–1.75; *p* = 0.04).

**Conclusion:**

The ultrasonographic method to evaluate thigh thickness was easily applicable and reproducible. The thickness of the quadriceps could predict rehospitalization or death, even in those patients without walking capacity—unable to perform gait speed and timed up and go tests. Additionally, the contractile index was associated with functional decline after 3 months of hospital discharge. This is a promising result, which highlights the bedside ultrasound of the quadriceps as a potential tool for the prognosis evaluation of bedridden hospitalized elderly patients.

## Introduction

Comprehensive geriatric assessment (CGA) is a valuable clinical instrument for detection of potentially health-threatening conditions in the elderly and it is independently associated with functional decline, rehospitalization, and death (1, 2). Objective measures of muscle function evaluation in CGA, such as gait speed and timed up and go test, are easily applicable and have good predictive capacity for clinical outcomes in this population (1, 3–6). However, the application of these tests is restricted to individuals with preserved walking ability, limiting its use in hospitalized patients. Therefore, there is a clear need for objective measures of muscle function evaluation that can be applied also for bedridden elders and that are equally capable of predicting rehospitalization and functional decline.

Point of care ultrasound, a fast, secure and non-invasive technique, has been applied in several clinical contexts, including the evaluation of changes in body composition associated with aging (7–9). This can be particularly useful to identify frail elders and to assess functional and mobility decline. Quadriceps muscle thickness and the proportion between the contractile segment of the thigh and the total anterior thigh thickness seem to be correlated with functional status and frailty (8) and, if correlated with gait speed and timed up and go test, would be a predictive variable applicable also for bedridden elders.

Considering the aforementioned reasons, this study was designed to evaluate the ability of quadriceps muscle thickness and the proportion between the contractile segment of the thigh and the total anterior thigh thickness, assessed with inhospital point of care ultrasound, to predict functional decline, rehospitalization, and death 3 months after hospital discharge, especially in bedridden patients.

## Materials and Methods

Cohort of elder patients hospitalized in a medium complexity unit from a teaching hospital in southern Brazil was conducted to test for clinical outcomes. The inclusion criteria were 65 years or older hospitalized from June to September 2016. The exclusion criteria were impossibility to do point of care ultrasound of the quadriceps muscle for clinical reason (e.g., bilateral suprapatellar amputation of lower limbs) and impossibility to do the handgrip strength test for clinical reason (e.g., amputation of upper limbs).

To evaluate the reproducibility of the ultrasound method, a pilot transactional study was designed up in a sample of 23 patients. A workshop directed to all research team was held by a point of care ultrasound specialist. This training program comprised a theoretical session on the principles and techniques of point of care ultrasound and a subsequential 2 h supervised practice course.

After this training, each examiner performed three ultrasound measurements of the participants’ dominant thigh. All examiners were blinded for previous ultrasound results obtained by the other research team members. Intra and interobserver agreement analysis were then tested as described in the Section “Statistical Analysis.”

### Variables Studied

Each participant included was submitted to an interview for collection of sociodemographic data (sex, age, race, schooling, marital status), cause of hospitalization, and CGA, including number of medications in use, Barthel scale for basic activities of daily living (10, 11), Lawton scale for instrumental activities of daily living (12), mini mental state exam (13), Charlson comorbidity index (14), calf circumference, gait speed, hand grip strength, and timed up and go test.

### Point of Care Ultrasound

Ultrasound measurements of the thigh were collected according to the previous description published by Agyapong-Badu et al. (8), using a portable ultrasound set (M-turbo Sonosite^®^) with a high frequency linear transducer (5–10 MHz) in preset for muscle evaluation on B-mode and getting transverse view of the anterior thigh. Ultrasound images were taken at a site two-thirds of the distance between the antero-superior iliac spine and the superior pole of the patella in the sagittal plane, with the participant resting in supine position.

The examiner, in all measures, should support the elbow so as not to exert pressure on the thigh site to be evaluated. To identify the patient’s dominant leg, the following question was asked: “With which leg do you usually kick a ball?” If the participant did not know how to answer, it was considered as dominant the leg ipsilateral to the dominant hand.

**Figure 1:**
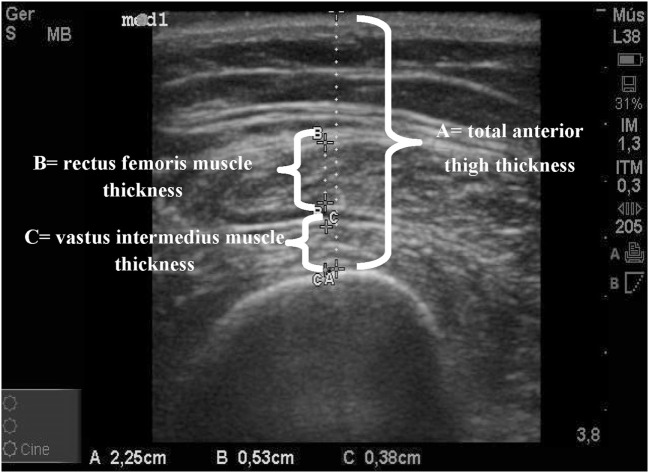
Ultrasound scan with measurements of the dominant thigh. A = total anterior thigh thickness in centimeters; B = rectus femoris muscle thickness in centimeters; C = vastus intermedius muscle thickness in centimeters; B + C = quadriceps muscle thickness in centimeters (attached).

The following measurements were made from B-mode: total anterior thigh thickness (measured in centimeters from the skin to the anterior border of the femur) and thickness of the quadriceps muscle (measured in centimeters from the internal borders of the rectus femoris muscle added to the measurement of the internal borders of the vastus intermedius muscle), thus excluding the perimuscular fascia, to measure only the contractile portion of the quadriceps muscle (Figure 1). Reference category for contractile index was ≥60% and for quadriceps thickness it was ≥1.2 cm.

The contractile index was obtained dividing the quadriceps muscle thickness by the total thickness of anterior thigh multiplied by 100. Values of 60 or over were defined as reference category after accuracy testing in the study’s sample.

### Follow-up

Three months after hospital discharge, a telephone call was made to verify rehospitalization or death information and to access basic activities of daily living score (Barthel scale) in the survivors.

### Statistical Analysis

The reproducibility of the ultrasound measurement of the thigh thickness was analyzed by the Bland–Altman method for interobserver agreement and the intraobserver agreement analysis was performed by the Generalized Estimating Equations (GEE) model with covariance matrix (robust estimator) and labor-independent correlation matrix.

Continuous variables were analyzed using the *T*-test and categorical variables using the chi-square test. Non-parametric equivalent tests were performed when non-normal data distribution was found. To evaluate correlations, the Spearman (categorical) and Pearson (continuous) tests were used, and the risks were calculated by univariate and multivariate analysis by Binomial Logistic Regression Model with robust estimator and Pearson chi-square as scale parameter method. Due to the high prevalence of the outcome in the reference categories, there was lack of power to perform multivariate analysis and it was performed as a sensitivity analysis. IBM SPSS Statistics for Windows, Version 19.0, 2010, Armonk, NY was the software used for all data analysis.

Considering the novel applicability of bedside ultrasound proposed in this pilot study, sample size calculation had to be based on a study that evaluated gait speed as functional predictive variable for clinical outcomes (15). Expecting a 50% incidence of rehospitalization in the exposed group (gait speed ≤ 0.6 m/s) and a relative risk of 2.5 (10) with 80% power to detect differences and alpha of 5%, the sample size needed was 90.

#### Ethics

This study was approved by the Hospital de Clínicas de Porto Alegre Ethics and Research Committee (Certificate of Presentation for Ethical Consideration number: 53460316800005327) and each participant signed an Informed Consent Form before inclusion.

## Results

During the period from June to September 2016, 115 eligible patients were consecutively invited to participate in this study. Of those, 100 signed the Informed Consent Form and were included. Of those, the first 23 had their ultrasound measurements performed by three independent examiners and these measurements were used to perform agreement analysis. Of all the 100 participants, only 1 could not have a follow-up assessment in 3 months (not found).

Table 1 shows the baseline participants’ characteristics: 54% were female, 92% were white, 16% were illiterate, 33% had between 1 and 4 years of schooling, and 47% were widowers. Pneumonia was the main cause of hospitalization in this study (32%).

**Table 1:** Baseline characteristics of studied population.

Characteristics	Cohort (*n* = 100)	

	*n* (%) or median ± SD	
**Sociodemographic**		
Women	54 (54)	
Age in years	78.6 (±9.3)	
**Race**		
White	92 (92)	
Black	5 (5)	
Other	3 (3)	
**Schooling**		
Illiterate	16 (16)	
1–4 years	33 (33)	
5–8 years	33 (33)	
>8 years	18 (18)	
**Marital status**		
Widower	47 (47)	
Married	34 (34)	
Single	13 (13)	
Separated	5 (5)	
Divorced	1 (1)	
**Cause of hospitalization**		
Pneumonia	32 (32)	
Decompensated heart failure	8 (8)	
Chronic obstructive pulmonary disease	11 (11)	
Urinary tract infection	15 (15)	
Dyspnea	14 (14)	
Delirium	3 (3)	
Diarrhea	3 (3)	
Others	17 (17)	
**Comprehensive geriatric assessment**		***n***
Barthel scale (BADL)	69.2 (±34.2)	100
Lawton scale (IADL)	13.2 (±5.0)	100
Mini Mental State Exam	20.6 (±4.9)	78
Charlson comorbidity index	3 (±2.0)	100
Gait speed (m/s)	0.63 (±0.29)	52
Timed up and go (s)	23.2 (±15.1)	52
Handgrip strength (kg/f)	21.2 (±8.9)	80
Calf circumference (cm)	32.3 (±4.8)	93
Number of medications in use	5.1 (±2.6)	100
**Ultrasonographic measurements**		
Total anterior thigh thickness (cm)	2.88 (±1.1)	97
Quadriceps thickness (cm)	1.65 (±0.69)	97
Contractile index (%)	57.9 (±13.6)	97

*BADL, basic activities of daily living; IADL, instrumental activities of daily living; hand grip strength, best out of three measurements in the dominant hand*.

### Method Reproducibility—Agreement Results

#### Intraobserver Agreement

Intraobserver analysis was performed using the model of Generalized Estimation Equations (GEE) for repeated measurements. Table 2 shows the results for the *total anterior thigh thickness* and *quadriceps thickness* values. There was no statistically significant difference between intraobserver measurements.

**Table 2:** Intraobserver agreement analysis.

Examiners		Median thigh thickness in centimeters (CI 95%)	*p*^a^	Median quadriceps thickness in centimeters (CI 95%)	*p*^a^
**Examiner 1**					
	Measure 1	2.71 (2.33–3.08)		1.49 (1.28–1.71)	
	Measure 2	2.76 (2.33–3.19)		1.44 (1.18–1.70)	
	Measure 3	2.79 (2.33–3.24)		1.47 (1.16–1.77)	
			0.585		0.771
**Examiner 2**					
	Measure 1	2.65 (2.26–3.05)		1.42 (1.20–1.64)	
	Measure 2	2.60 (2.17–3.03)		1.52 (1.27–1.77)	
	Measure 3	2.68 (2.24–3.11)		1.57 (1.33–1.82)	
			0.098		0.079
**Examiner 3**					
	Measure 1	2.76 (2.40–3.12)		1.45 (1.21–1.70)	
	Measure 2	2.74 (2.39–3.09)		1.46 (1.22–1.71)	
	Measure 3	2.79 (2.41–3.18)		1.50 (1.24–1.77)	
			0.517		0.361

*^a^Chi-square Wald’s test. Generalized Estimating Equations (GEE)*.

*Examiners 1 and 2, geriatric residents 1 and 2; examiner 3, point of care ultrasound specialist*.

#### Interobserver Agreement

**Figure 2:**
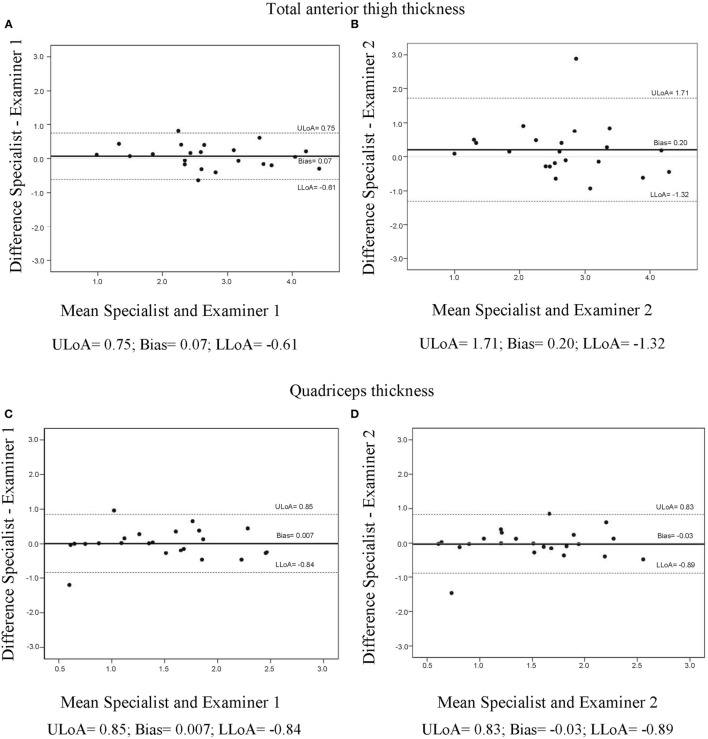
Bland–Altman interobserver agreement analysis for total anterior thigh thickness **(A,B)** and quadriceps thickness **(C,D)**. ULoA, upper limit of agreement; LLoA, lower limit of agreement (attached).

Figure 2 shows the plot of interobserver agreement obtained through the Bland–Altman method. The mean bias was close to zero in analysis for *total anterior thigh thickness* (0.07 for specialist versus examiner 1 and 0.20 for specialist versus examiner 2) and in the analysis for *quadriceps thickness* (0.007 for specialist versus examiner 1 and 0.03 for specialist versus examiner 2). The distribution of the differences was homogeneous and within the limits of agreement of two SDs in all four performed analyses.

### Clinical and Predictive Results

#### Correlations with CGA Tests

Significant correlations were found between the ultrasound variables *quadriceps thickness* and *contractile index* and measures of CGA muscle function evaluation, as shown in Table 3.

**Table 3:** Correlation between ultrasonographic variables and muscle function tests from CGA.

Ultrasonographic measurements	Muscle function tests							
	Gait speed		TUG		Handgrip		Calf circumference	
	Spearman	*p*	Spearman	*p*	Spearman	*p*	Spearman	*p*
Total thigh thickness	0.054	0.707	(−) 0.012	0.935	0.144	0.209	0.786	<0.001
Quadriceps thickness	0.317	0.023	(−) 0.297	0.034	0.411	<0.001	0.728	<0.001
Contractile index	0.414	0.003	(−) 0.434	0.001	0.432	<0.001	0.041	0.702

*CGA, comprehensive geriatric assessment; TUG, timed up and go test*.

#### Clinical Outcomes

After 3 months of hospital discharge, the incidence of functional decline was observed in 51.2% of the sample, rehospitalization in 34%, and death in 17%. None of the sociodemographic and physical function variables, when categorized, were associated with the outcome of rehospitalization or death. The only continuous variable that was associated with this outcome was the Barthel scale for basic activities of daily living.

There was a statistically significant association between decreased contractile index (<60%) and functional decline 3 months after hospital discharge (RR 1.35; CI 95% 1.10–1.65; *p* = 0.003) (Table 4). After multivariable analysis considering age and sex as covariates, it did not remain statistically associated with functional decline possibly by lack of power. Similar results were found for gait speed and timed up and go tests (Table S1 in Supplementary Material).

**Table 4:** Risk for clinical outcomes by ultrasonographic and mobility tests variables.

	Quadriceps thickness ≤ 1.2 cm			Contractile index ≥ 60%		
	RR	CI 95%	*p*	RR	CI 95%	*p*
Rehospitalization or death						
All participants (*n* = 100)	1.24	1.01–1.5	0.04	1.13	0.9–1.4	0.2
Bedridden (*n* = 48)	1.34	1.02–1.75	0.04	1.24	0.9–1.6	0.1
Functional decline	1.1	0.8–1.4	0.3	1.35	1.1–1.6	0.003

Additionally, *quadriceps thickness* was associated with rehospitalization or death 3 months after hospital discharge (RR 1.24; CI 95% 1.01–1.5; *p* = 0.04). Adjusted analysis for sex and age showed a tendency for significant association (RR 1.2; CI 95% 0.9–3.5; *p* = 0.08). It is important to highlight the results considering only bedridden elderly. In this group of patients unable to perform mobility tests, the risk for rehospitalization or death was 30% higher when *quadriceps thickness* was less than 1.2 cm (RR 1.34; CI 95% 1.02–1.75; *p* = 0.04) (Table 4). Gait speed and timed up and go test were not associated with greater risk for rehospitalization or death (Table S2 in Supplementary Material).

## Discussion

The bedside ultrasound method tested in this study for the evaluation of the anterior thigh of hospitalized elderly patients was easily applicable with good intra and interobserver agreement. It was also evidenced that both *quadriceps thickness* and the *contractile index* had significant correlation with gait speed, timed up and go, and handgrip strength. Beyond that, low quadriceps thickness predicted rehospitalization or death 3 months after hospital discharge and, as novelty and clinically relevant information, this finding remained significant among bedridden elderly. Associations tested were not significant after adjustments for age or sex, possibly because of lack of power to test for counfounding factors.

Previous studies have shown that reduction in skeletal muscle mass and strength occur throughout aging, even in the healthy elderly, and highlight the need for continued research into this age-related problem (16). Previous study also showed association between anthropometric measures, physical activity patterns, and outcome of mobility and falls (17). However, most of the available research findings regarding body composition in older patients were based in dual-energy X-ray absorptiometry (DXA), computed tomography (CT), or magnetic resonance imaging (MRI) as the measurement method (18–21). Given the relatively high cost of CT and MRI, the radiation exposure of CT and DXA, and the attractive qualities of ultrasound, such as increasing availability in hospital setting, low cost, easy transport, and radiation-free, the bedside ultrasound method to evaluate the anterior thigh should be considered in the prognostic evaluation of hospitalized elders as an alternative, safer, easily applicable and validated method.

More recently, the number of studies evaluating different clinical applications for bedside ultrasound has increased (7, 8). Mourtzakis and Wischmeyer (22) have applied bedside ultrasound to access body composition in the elderly population and describe the method as a useful tool to measure the muscle mass and the changes in muscle tissue. However, as novelty, this is the first study to validate a bedside ultrasound method of anterior thigh of hospitalized elders, including those unable to walk, that can predict rehospitalization or death as well as functional decline.

Several clinical conditions can compromise the global health status of a hospitalized elderly patient, increasing the inhospital and post-discharge morbimortality. The frailty syndrome is one of those. Elderly patients with this syndrome have lower functional reserve and higher incidence of negative outcomes such as rehospitalization and functional decline (23, 24). In the Cardiovascular Health Study (24), frailty was defined as the presence of three or more of the following five clinical criteria: weakness, poor endurance and energy, slowness, low physical activity level, and unintentional weight loss. This frailty phenotype and the accumulation of deficits throughout life (25) were able to predict falls, loss of mobility, functional decline in activities of daily living, as well as risk for hospitalization and death. However, it is composed by association of distinct subjective and objective clinical variables, some difficult to obtain, especially in hospital scenarios and in bedridden patients. Bedside ultrasound measurements of the anterior thigh, simple and easy clinical variables to obtain, tested as an alternative method to assess important prognostic factors in this study, were also correlated with mobility tests, functional decline and, most importantly, significantly associated with clinical outcomes in this sample of hospitalized elderly patients.

Gait speed and timed up and go test, objective measures of muscle function evaluation present in CGA, are easily applicable and have good predictive capacity for clinical outcomes in this population (1, 3–6). However, the uses of these tests are limited in hospitalized bedridden patients, a usually vulnerable population in which a physical objective evaluation can provide very useful clinical information. In this way, the most relevant result evidenced by this study according to the authors is that in bedridden hospitalized elderly patients, a low quadriceps thickness increased the risk for rehospitalization or death and, in this way, was able to additionally distinguish different prognosis profiles even among those vulnerable individuals, characterizing its novel clinical applicability. As far as we could investigate, this is the first study to validate a bedside ultrasound method of thigh evaluation in hospitalized elders and to present an objective evaluation of muscular functionality of hospitalized elderly unable to walk that can predict rehospitalization or death. This results brings a potential benefit of point of care ultrasound in the evaluation of hospitalized elderly patients, and may add significant value to the inhospital and post-hospital care of these patients, especially for identifying those with higher risk for negative outcomes.

Furthermore, low contractile index, a novel variable proposed and tested for the first time in this study, was able to predict functional decline 3 months after hospital discharge. The same was not observed for the quadriceps thickness variable. Because it represents the contractile segment of the anterior thigh over its entire thickness, and not just the isolated measure of quadriceps muscle thickness, the contractile index seems to better reflect the muscle reserve of the lower limb in comparison with the thickness of the quadriceps itself. In a geriatric care context, the capacity of contractile index to predict functional decline is a promising result and makes this a potential tool for the evaluation of hospitalized elderly patients. And, as authors hypothesize for future studies, it would help select potentially vulnerable patients for individualized care planning and clinical actions to prevent further functional decline.

The main limitation of the present study was the lack of power to test for independent associations regarding important theoretical confusion variables. Additionally, it is important to emphasize baseline characteristics that can interfere with external validity as the sample was provenient from a medium complexity hospital. Therefore, further studies with bigger sample size and longer follow-up period may be required for a more accurate assessment of the ability of ultrasound measurements of the anterior thigh to predict rehospitalization or death, functional decline, and to test its predictive capacity as a clinical instrument to be used as inclusion criteria in intervention studies.

In conclusion, the ultrasound method tested was easily applicable and reproducible. And, most importantly, it was able to demonstrate that the lower the contractile segment of the anterior thigh the greater the functional decline 3 months after discharge. Moreover, the quadriceps thickness measured by bedside ultrasonography was able to predict rehospitalization or death in all hospitalized elderly groups, including those bedridden elders, who were unable to walk. These results highlight this ultrasound method as a potential tool for prognostic assessment of hospitalized elderly, especially those unable to perform mobility tests.

## Ethics Statement

This study was carried out in accordance with the recommendations of the “Resolution number 466 of the National Health Council of the Brazilian Ministry of Health” with written informed consent from all subjects. All subjects gave written informed consent in accordance with the Declaration of Helsinki. The protocol was approved by the “Hospital de Clínicas de Porto Alegre Ethics and Research Committee.”

## Author Contributions

RM: research protocol development and planning; protocol conduction and supervision; inclusion of participants; data analysis; interpretation of results; manuscript writing; and revisor leader. AG: research protocol development and planning; protocol conduction and supervision; inclusion of participants; bedside ultrasound examiner; data typing; interpretation of results; and manuscript writing. RO: inclusion of participants; bedside ultrasound examiner; data typing; interpretation of results; and manuscript writing. AT: research protocol development and planning; inclusion of participants; bedside ultrasound specialist; data analysis; interpretation of results; and manuscript writing and manuscript revision. RC: research protocol development; data analysis; interpretation of results; and manuscript writing. EM: research protocol development; interpretation of results; manuscript writing; and manuscript revisor.

## Conflict of Interest Statement

The authors declare that the research was conducted in the absence of any commercial or financial relationships that could be construed as a potential conflict of interest. The reviewer, PB, and the handling editor declared their shared affiliation, and the handling editor states that the process nevertheless met the standards of a fair and objective review.
