# Supplementary material for: Bedside Ultrasound of Quadriceps to Predict Rehospitalization and Functional Decline in Hospitalized Elders
Source: Front Med (Lausanne). 2017 Jul 31;4:122. doi: 10.3389/fmed.2017.00122 (PMC5535297; doi:10.3389/fmed.2017.00122)
Supplement: Supplementary file 1 [file Table_1.DOCX]

| **Table S1** Risk for functional decline by ultrasonographic and mobility tests variables | | | |
| --- | --- | --- | --- |
|  |  |  |  |
|  |  |  |  |
|  | RR | IC(95%) | P |
| Gait Speed ≤ 0.6m/s | 1.5 | 1.1-1.9 | 0.004 |
| TUG ≥ 20s | 1.5 | 1.1-1.9 | 0.006 |
| Quadriceps thickness | 1.1 | 0.8-1.4 | 0.3 |
| Contractile index | 1.35 | 1.1-1.6 | 0.003 |
| CGA: Comprehensive geriatric assessment; TUG: Timed up and Go test | | | |
|  |  |  |  |

Supplementary Data
