# Supplementary material for: Bedside Ultrasound of Quadriceps to Predict Rehospitalization and Functional Decline in Hospitalized Elders
Source: Front Med (Lausanne). 2017 Jul 31;4:122. doi: 10.3389/fmed.2017.00122 (PMC5535297; doi:10.3389/fmed.2017.00122)
Supplement: Supplementary file 2 [file Table_2.DOCX]

Supplementary Data

| **Table S2** Risk for Rehospitalization or Death by ultrasonographic and mobility tests variables | | | |
| --- | --- | --- | --- |
|  |  |  |  |
|  | RR | IC(95%) | P |
| Gait Speed ≤ 0.6m/s | 1.2 | 0.9-1.5 | 0.2 |
| TUG ≥ 20s | 1.2 | 0.9-1.6 | 0.2 |
| Quadriceps thickness | 1.24 | 1.01-1.5 | 0.04 |
| Contractile index | 1.13 | 0.9-1.4 | 0.2 |
| CGA: Comprehensive geriatric assessment; TUG: Timed up and Go test | | | |
|  |  |  |  |
